# Clinical identification and endodontic management of furcation canals: a case series

**DOI:** 10.1590/0103-6440202304817

**Published:** 2023-03-06

**Authors:** Pierre Kuoch, Martin Biosse Duplan, Fleur Berès, Éric Bonte, Cauris Couvrechel

**Affiliations:** 1 Service de Médecine Bucco-Dentaire, Hôpital Bretonneau (AP-HP), Paris, France; 2 Pratique privée, Paris, France; 3 UFR d’Odontologie - Montrouge, Université Paris Cité, Paris, France

**Keywords:** Endodontics, Endo-periodontal lesions, Furcation, Periodontal disease

## Abstract

In the case of endodontic infection, the presence of furcation canals can be at the origin of a periodontal lesion located in the furcation. The furcation being very close to the marginal periodontium, this type of lesion can be favorable to the genesis of an endo-periodontal lesion. These furcation canals are lateral canals located on the pulp chamber floor and constitute one of the many physiological communication pathways between endodontic and periodontal tissues. These canals are most often difficult to localize, shape, and to fill because of their small diameter and length. The disinfection of the pulp chamber floor with sodium hypochlorite solution may contribute to the disinfection of furcation canals when they are not identified, shaped, and/or filled. This case series illustrates the endodontic management of visible furcation canals associated with an endo-periodontal lesion. These furcation canals had a large diameter which allowed their identification during the endodontic treatment.

## Introduction

Studies evaluating the success rate of endodontic treatments often reveal disappointing results in general practice ^1^. The causes of failure are multiple and multifactorial. The residual reservoir of bacteria and/or toxins in the endodontic system is a major factor and is often due to a lack of accurate knowledge of the endodontic anatomy. The omission of second mesiobuccal canals and subsequent endodontic failure have been frequently reported ^2^ but there is another important entity to be aware of in order to minimize failure: the furcation canal.

Furcation canals are classified as lateral canals located at the pulp chamber floor or on the coronal aspect of a root canal of premolars and molars ^3^ ^,^ ^4^. They are physiological communication pathways between the endodontic and the periodontic tissues alongside with apical foramen and dentinal tubules ^5^. Although rarely identified clinically, they are an important anatomic entity, as they can be responsible for the presence of an inter-radicular lesion ^3^ and an endo-periodontal lesion. A higher prevalence of furcation involvement (horizontal extent ≥ 3 mm) has been observed on mandibular molars with periapical lesions ^6^. Furcation involvement may decrease the prognosis of the tooth, particularly in patients with periodontitis ^7^. In practice, the identification of these often-invisible canals is difficult or fails without a magnification lens. Their small diameter and their coronal situation make their disinfection and filling difficult.

## Case Report

### Case #1:

A 50-year-old non-smoking woman with no past medical/surgical background (ASA 1) was referred for endodontic treatment of the right mandibular second molar (#47). Her dentist had initiated the endodontic treatment 5 months earlier. The tooth presented an irreversible pulpitis following a secondary tooth decay under a composite restoration and a coronal fracture of the lingual aspect. The endodontic treatment had not been completed following the fracture of an endodontic file in the mesiobuccal root canal.

**Figure 1:**
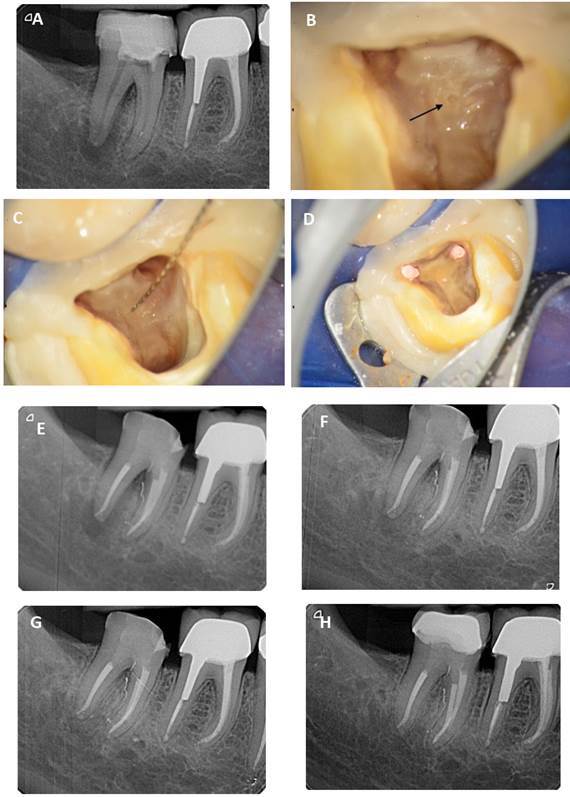
(A) Preoperative intraoral X-Ray focused on tooth #47; (B) Furcation canal foramen indicated by the arrow; (C) Furcation canal after K 06 file in situ; (D) Endodontic filling of the mesiobuccal, mesiolingual, distal, and furcation canals; (E) Postoperative intraoral X-Ray focused on tooth #47. Endo-restorative continuum implemented with a resin composite-build up in the access cavity; (F) Intraoral X-Ray at 3 months postoperative. (G) Intraoral X-Ray at 8 months postoperative; (H) Intraoral X-Ray at 12 months postoperative. Healing of periapical and furcation lesions.

Clinical examination revealed negative tests of axial and lateral percussion, lingual palpation, and bite. However, vestibular palpation was painful. Periodontal probing revealed an attachment loss that involved the entire buccal surface and the mesial buccal angle of the distal root, with a pocket depth (12 mm) reaching the apex root. The examination showed the presence of a class 2 inter-radicular lesion ^8^. There was no bleeding on probing and no periodontal recession around the tooth. The gingival plaque index ^9^ was 1 and tooth mobility was physiological (stage 1) ^10^. The patient attended a periodontal maintenance program following treatment of a localized periodontitis (stage I / grade B ^11^). X-Ray examination revealed a periradicular and periapical radiolucency of 6.5 mm in diameter at the distal root, as well as a radiolucency below the furcation (Figure 1A). The tooth showed no radiological evidence of root perforation or resorption. The diagnosis was a grade 2 endo-periodontal lesion without loss of root integrity in a patient with periodontitis ^7^. It was decided to perform endodontic treatment of tooth #47. The patient was advised that periodontal treatment may be required in a second step.

After local anesthesia, the root surface was inspected under an operating microscope (OPMI pico, Zeiss, Oberkochen, Germany) and revealed no evidence of root fracture. A rubber dam was set up, isolating the second and first right mandibular molar. The provisional crown was removed, and the rubber dam was sealed. The endodontic treatment was performed following the usual steps of root canal localization, scouting, cleaning, and shaping (Protaper Gold, Dentsply Sirona, York, Pennsylvania, United States of America) under abundant and renewed flushing of a 3% sodium hypochlorite solution (Sodium Hypochlorite Solution 3%, Vista Dental Products, Racine, Wisconsin, United States of America). The separated file could be "bypassed" and removed during irrigation. The inspection of the pulp chamber floor, using the operating microscope, revealed a foramen in its middle. A 06-size K-file (K-Files Ready Steel, Dentsply Sirona, York, Pennsylvania, United States of America) was inserted into the foramen. The use of an electronic apex locator (Root ZX mini, Morita, Dietzenbach, Germany) confirmed the patency of this canal with the periodontium (Figure 1B and 1C). A final rinse of root canals was performed with an EDTA 17% solution (EDTA 17% Solution, Vista Dental Products, Racine, Wisconsin, United States of America) for 1 minute and then 3% sodium hypochlorite for 10 minutes. These solutions were activated with a sonic handpiece (Endoactivator, Dentsply Sirona, York, Pennsylvania, United States of America). Vertical movements with the ISO 06 K-file were performed through the furcation canal to renew the sodium hypochlorite solution while avoiding the widening of the canal. After the final rinsing with saline, the final drying of the canals was carried out using sterile paper points (Paper Points, VDW, Munich, Germany). Microsuction (0.48 mm Capillary Tips, Ultradent, South Jordan, Utah, United States of America) was used to dry the pulp chamber floor and the furcation canal. The root canals were filled with gutta-percha (Gutta Autofitt, SybronEndo, Orange, California, United States of America) using a warm vertical compaction technique in several waves (Elements Free Down Pack, SybronEndo, Orange, United States of America; 04 Buchanan Heat plugger, SybronEndo, Orange, California, United States of America). The gutta-percha cones were previously disinfected in a 3% sodium hypochlorite solution, dried, and then impregnated with zinc oxide (ZnO)-eugenol-based endodontic filling sealer (Pulp Canal Sealer, Kerr, Orange, California, United States of America). Compaction was completed using the Mac Spadden technique (Gutta Condendor, Dentsply Sirona, York,Pennsylvaniae, United States of America). ZnO-eugenol endodontic cement was placed using a new ISO 06 K-file (K-Files Ready Steel, Dentsply Sirona) in the furcation canal and in excess on the pulpal floor. Gutta-percha, heated by thermomechanical compaction (Gutta Condensor, Dentsply Sirona), was placed on the pulpal floor and compacted with an endodontic plugger. After cooling and hardening, the gutta-percha was removed from the pulp chamber with a round tungsten carbide bur (EndoTracer, Komet, Lemgo, Germany) without the air-water spray, leaving only the inlets of the four canals blocked, including the furcation canal (Figure 1D). The floor and walls of the access cavity were cleaned and sandblasted. The access cavity was filled with a composite restoration (EverX, GC, Tokyo, Japan; G-aenial Anterior, GC, Tokyo, Japan) using a three-step adhesive protocol (etching: Uni-Etch 37%, Bisico; primer and adhesive: Optibond FL, Kerr, Orange, California, United States of America) in order to obtain coronal sealing in the session. A temporary crown was made in the session and then provisionally sealed. Attention was paid to the strict removal of excess temporary sealing cement. Post-operative retro-alveolar radiography revealed the filling of the furcation canal (Figure 1E). Cement extrusion through a lateral canal on the mesial root was also observed.

At three months postoperatively, the tooth was asymptomatic, and the clinical examination was normal. The periapical radiological bone lesion at the distal root decreased in size. Periodontal probing had decreased to 3 mm without a gingival recession. The periodontal lesion showed signs of clinical and radiographic healing (Figure 1F). At eight months postoperatively, the tooth remained asymptomatic and a 2 mm radiolucent area between the roots persisted (Figure 1G). It was then decided to make the final prosthetic restoration. At twelve months postoperatively, periodontal probing was stable (3 mm) with no gingival recession. Periradicular and inter-radicular bone lesions had completely resolved (Figure 1H).

### Case #2:

A 45-year-old non-smoking woman with no past medical/surgical background (ASA 1) was referred for endodontic treatment of the right mandibular second molar (#47). A composite restoration was set 7 months ago. The patient complained of swelling facing tooth #47, 2 months after the restorative procedure.

**Figure 2:**
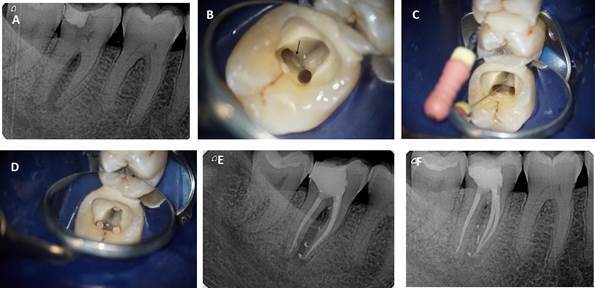
(A) Preoperative intraoral X-Ray focused on tooth #47; (B) Furcation canal foramen indicated by the arrow; (C) #06 K-file instrument inserted into the furcation canal; (D) Endodontic filling of the mesiobuccal, mesiolingual, distal and furcation canals; (E) Postoperative intraoral X-Ray focused on tooth #47; (F) X-Ray at 12 months postoperative: healing of periapical and furcation lesions.

Clinical examination revealed the presence of a sinus track facing the buccal mucosa of the tooth #47. The pulp sensitivity with cold and electrical tests was negative. Percussion and palpation tests were also negative. Periodontal probing gave a periodontal pocket of 5 mm attachment loss facing the buccal aspect of the mesial root, with bleeding on probing. The gingival plaque index ^9^ was 2 and tooth mobility was physiological (stage 1) ^10^. Intraoral radiographic examination revealed a periradicular and periapical radiolucency of 7.2 mm in diameter between the distal and mesial roots and under the furcation area (Figure 2A). The examination revealed the absence of an iatrogenic and/or traumatic event. The diagnosis was a chronic periapical abscess associated with a grade 1 endo-periodontal lesion without loss of root integrity in a patient without periodontitis ^12^. It was decided to perform a root canal treatment of tooth #47. The patient was advised to follow an etiologic periodontal treatment.

All the endodontic management procedures of isolation, access cavity, shaping, cleaning, and obturation were performed following the same protocol as described in the Case #1.

During treatment, the pulp chamber floor was inspected under the operating microscope and revealed a foramen on the pulp chamber floor. This foramen was located in a mesial position on the pulp chamber floor. A 06-size K-file (K-Files Ready Steel, Dentsply Sirona) was glided into the foramen (Figure 2B to 2D). The use of an electronic apex locator (Root ZX mini, Morita) confirmed its patency with the periodontium and confirmed also it was not a mesio-central canal by its small length. The furcation canal was shaped with a rotary instrument (Protaper Gold, Dentsply Sirona) until the F1 finishing file. The patency was controlled with an ISO 06 K-file. The disinfection was processed as the others canals and solutions were activated with a sonic handpiece (Endoactivator, Dentsply Sirona). Obturation of the furcation canal was done with heated gutta-percha by warm vertical compaction technique: a gutta-percha point (Protaper Gold F1 gutta percha points, Dentsply Sirona, York, Pennsylvania, United States of America) with ZnO-eugenol-based endodontic sealer was introduced in the furcation canal, cutted at the entry and heated before compaction. After cleaning of the access cavity, a coronal restoration was performed with a composite restoration (EverX Flow, GC; G-aenial Anterior, GC) using a three-step adhesive protocol (etching: Uni-Etch 37%, Bisico; primer and adhesive: Optibond FL, Kerr) to ensure an immediate coronal sealing. The postoperative radiograph showed the obturation of main canals, furcation, and lateral canals (Figure 2E).

At two weeks postoperative, the sinus track disappeared. At six months postoperative, the tooth was asymptomatic. The periodontal probing and the clinical examination were normal. It was then decided to make the final ceramic prosthetic restoration (IPS e.max, Ivoclar Vivadent, Shaan, Liechtenstein). The radiographic examination showed a complete endo-periodontal healing at twelve months postoperative (Figure 2F).

## Discussion

Furcation canals are physiological communication pathways between the pulp and the periodontium at the furcation level. They are often incriminated when an inter-radicular radiolucency of endodontic origin is observed in multi-rooted teeth. Their presence, incidence, location, and diameter are well reported in experimental studies ^13^ ^,^ ^14^ but furcation canals are often clinically invisible.

The floor of pulp chamber has foramina ranging from 7 to 34 µm in diameter ^15^. The number of foramina can vary from zero to more than twenty on a molar. Foramina involving both the pulp chamber floor and the furcation root surface are observed in 36% of maxillary first molars, 12% of maxillary second molars, 32% of mandibular first molars, and 24% of mandibular second molars ^16^. Mandibular teeth have a higher incidence (56%) of foramina involving both the pulp chamber floor and the furcation root surface compared to maxillary teeth (48%). On the other hand, 46% of the mandibular first molars have lateral canals opening into the inter-radicular region, but the origin of the lateral canal may be in the coronal third of the main canal or on the pulp chamber floor. From these 46%, the frequency of a single direct furcation canal extending from the pulp chamber floor to the furcation region is about 13%. In these situations, the diameter of the furcation canal may be larger (240 µm ^15^ and can be clinically detected ^14^.

Another ex vivo study observed that 2.8% of mandibular molars and 0.3% of maxillary molars have furcation canals communicating between the pulp chamber floor and the furcation area ^17^. This difference with previous studies could be explained by the sampling and research method. Moreover, in the studies ^17^ ^,^ ^18^ that showed a very low incidence of furcation canals, a difference is made between a furcation canal (continuous canal with 2 portals of exit/entrance that connect the endodontic and the periodontic tissues) and a diverticulum (interradicular canal without patency originating either at the pulp chamber floor or the furcation surface). In fact, diverticula (4.3%) are more frequently observed than furcation canals (1.4%) on all molars combined.

When there is a single furcation canal, its foramen is located in the center of the pulp chamber floor (57.1%) but may also have a more mesial position (28.5%) on the floor and more rarely a distal position (14.4%). The average length from the pulp chamber floor to the furcation region is 3.9 mm ^14^. Branching of the furcation canal may be present within the dentin and/or cementum, which explains the greater presence of foramina at the furcation level than at the pulpal chamber floor level ^15^ ^,^ ^19^. It should also be noted that most (70%) of the foramina on the floor do not open in the interradicular area because they are closed by the cementum or by an ostecementum-type tissue ^3^ ^,^ ^16^. They may be exposed during aggressive root planning, external root resorption ^6^, or severe damage to the pulp chamber floor for diverticula originating from the furcation area. By the possibility of apposition of secondary or tertiary dentin or cement, the frequency of furcation canals could be higher in younger teeth ^18^.

The observed foramen on the pulpal chamber floor in ex vivo studies did not contain any vessels, nerves, or connective tissue after 5 minutes of action of sodium hypochlorite solution ^15^ ^,^ ^20^. This suggests that a chemical disinfection performed during all the treatments by sodium hypochlorite solution retained by the walls of the access cavity may contribute to the disinfection of furcation canals. It is then highly recommended to perform thorough chemical cleaning and disinfecting of the pulp chamber floor in order to minimize treatment failure ^18^. Without adequate chemical disinfection of the pulp chamber floor, successful endodontic treatment could be compromised in up to 10% of the cases ^17^ ^,^ ^18^.

Several methods have been suggested for the filling of furcation canals after chemical disinfection: by compressing ZnO-eugenol cement with a cotton pellet ^21^ or by vertically compacting heated gutta-percha ^22^ on the pulp chamber floor. There is no scientific evidence in favor of systematizing endodontic sealing of the pulp chamber floor by endodontic cement compaction. Indeed, it is impossible to properly clean a lateral canal without instrumenting it. Nor does its filling guarantees the absence of infection. It has been shown that despite the filling of the lateral canals, necrotic pulpal tissue mixed with the endodontic filling material could be the cause of endodontic failure ^23^. Nevertheless, attention should be paid to the presence of these often-invisible furcation canals during each root canal treatment of multi-rooted teeth, especially when an inter-radicular radiolucency is observed on the periapical radiograph.

A coronal sealing by bonding or sealing a coronal-root restoration is mandatory to prevent leakage of the endodontic system. In addition, attention should be paid to properly cleaning the pulp chamber after the root canal obturation due to the negative interaction of all endodontic sealers with bonding protocols ^24^.

## Conclusion

In these clinical cases, the important diameter of the furcation canal allowed exceptionally to highlight it during the treatment, to carry out a chemo-mechanical disinfection, and to fill it with gutta-percha and endodontic sealer like any root canal. Healing was obtained without the need for local periodontal therapy due to the endodontic origin of the endo-periodontal lesion.

Furcation canals play a significant role in the rate of success or failure of endodontic treatment. The chemical cleaning of the pulp chamber floor and the coronal sealing are important to prevent infection of endodontic origin.
